# Effects of mixed application of avermectin, imidacloprid and carbendazim on soil degradation and toxicity toward earthworms

**DOI:** 10.1038/s41598-023-41206-1

**Published:** 2023-08-29

**Authors:** Xiaoyu Liang, Yufei Li, Zhao Zheng, Fang Tian, Yannan Du, Ye Yang, Meng Wang, Yu Zhang

**Affiliations:** 1 Sanya Nanfan Research Institute, College of Plant Protection, Hainan University, Haikou, Sanya, China; 2 Hainan Institute for Food Control, Key Laboratory of Tropical Fruits and Vegetables Quality and Safety for State Market Regulation, Haikou, China

**Keywords:** Ecology, Environmental sciences

## Abstract

The application of pesticides in mixtures often exerts multiple pressures on agricultural soils in the short term. Therefore, it is necessary to assess the effects of mixed application on the environmental behavior and ecotoxicity of pesticides in soil. In this study, we assessed the effects of three common pesticides through mixed application on soil degradation and toxicity toward the earthworm *Eisenia fetida*. Compared with the degradation half-lives (DT50) the single pesticide, the DT50 values of avermectin, imidacloprid and carbendazim in the binary mixtures were similar. However, their DT50 values in the ternary mixtures were approximately 1.5 times longer than those in the individual applications, enhancing their stable in soil after two or three applications. The ternary mixtures of the pesticides showed significantly synergistic toxicity toward *E. fetida*, while their binary mixtures exhibited a changing interaction throughout the entire effect level range. The ternary mixtures activated higher SOD and CAT activities in *E. fetida* than the individual treatments, confirming their synergistic effects. By conducting avoidance tests with *E. fetida*, ternary toxic interactions were effectively assessed within a relatively short testing period. In summary, the three pesticides in ternary mixtures exhibited longer degradation half-lives and synergistic toxicity toward earthworms compared to individual or binary mixtures.

## Introduction

Soils underpin agricultural planting systems and experience numerous anthropogenic pressures, but we know little about the effects of these pressures on soils when they act together^1^. As important agrochemicals, pesticides in soils are widespread contaminants in agricultural fields and often coexist as a mixture^2^. Especially in the past few decades, with an increasing number of pesticides being released in the market, agricultural practices employ pesticides applied at lower doses but often in mixtures. The risk assessment of these mixtures becomes challenging because of their complex and variable combinations. In addition, the current risk assessment framework focuses on single chemicals, which cannot predict the actual toxicity of pesticide mixtures because of the joint effects in a mixture system^3^. Several pesticide mixtures have been found to not only exhibit elevated levels of pesticide residues but also to have synergistic impacts on the toxicity of soil invertebrates^4–6^. Applicators need to be concerned about the side effects of pesticide mixtures because of the possible combined joint effects.

As commonly used pesticides, avermectin, imidacloprid and carbendazim are widely applied to crops worldwide. Avermectin and imidacloprid are often used in combination to control aphids. These two insecticides are also often used in combination with carbendazim to cooperatively control the pests and diseases of soybean and wheat in China. Previous studies have documented the environmental behaviors of these pesticides individually in soil^7–9^. However, their effects as mixtures have not been investigated, despite their potential for co-occurrence in agricultural soils. In addition, these three pesticides showed a degree of toxicity toward nontarget organisms in soil. Avermectin, a macrolide insecticide, has toxic effects on the survival and reproduction of soil-dwelling invertebrates^10^. Imidacloprid, a neonicotinoid insecticide, besides of being toxic to the same endpoints as avermectin, induces oxidative stress and DNA damage in earthworms^11^. Carbendazim, a benzimidazole fungicide, shows moderately acute toxicity and genotoxicity toward earthworms^12^. Due to their different toxicities and occurrence in the same soil environment, their joint toxicity toward nontarget organisms in soil needs to be studied.

The earthworm *Eisenia fetida* is widely used for assessing the ecological risks of toxic chemicals in soil^13^. The typical ecological risk assessment framework of toxic substances focuses on acute toxic effects, e.g., mortality and biomarkers, which are prognostic and responsive to evaluate the stress of toxic chemicals^14^. The joint toxicity of chemicals calculated by the combination index (CI)-isobologram could well reveal the acute toxic interaction of mixtures^15^. However, the acute toxicity test needs to assess the survival of high-quality adult worms within two weeks^16^. Thus, there is a need to perform a simple, fast and reliable test for evaluating the possible risk of chemicals to soil organisms. Earthworm chemoreceptors are highly sensitive to chemicals in soil, so the avoidance test is a promising candidate for a short-term screening test^17^.

Considering these concerns, the target of this study on avermectin, imidacloprid and carbendazim was therefore to (i) determine the degradation half-life of their binary and ternary mixtures in soil, (ii) determine the toxicity of their binary and ternary mixtures towards earthworms *E. fetida*, and (iii) explore an assessment method for toxicity of pesticide co-occurrence toward earthworms.

## Materials and methods

### Chemicals and reagents

The standards of avermectin, imidacloprid and carbendazim were provided by Dr. Ehrenstorfer, GmbH (Augsburg, Germany). Stock standard solutions of avermectin and imidacloprid, with a concentration of 1000 mg/L, were prepared using methanol as the solvent. On the other hand, the stock standard s solution of carbendazim, with a concentration of 200 mg/L, was prepared using acetonitrile as the solvent. These three stock solutions were diluted with methanol to obtain a series of calibration standards (0.01, 0.02, 0.05, 0.1, 0.2, 0.5, 1 and 2 mg/L). All solutions were stored at 4 °C away from light before use. Avermectin (95% purity), imidacloprid (96% purity) and carbendazim (97.6% purity) were purchased from Anhui Huaxing Chemical Industry Co., Ltd. (Anhui, China). Avermectin (1.8%, emulsion), imidacloprid (10%, emulsion), and carbendazim (50%, wettable powder) were sourced from Zhejiang Sega Science and Technology Co., Ltd., Qingdao Zhengdao Pharma Co., Ltd., and Shanghai Shenlian Biotechnology Co., Ltd., respectively.

### Test organisms

Adults of the earthworm *E. fetida* weighing approximately 400 mg with well-developed clitella were purchased from Hainan Star Nongfu Ecological Technology Co., Ltd. (Hainan, China). Earthworms were cultured in the laboratory at 20 °C in artificial soil according to OECD guidelines^18^.

### Field trial

The residue study of these three pesticides and their mixtures was carried out in the field at the experimental base at Hainan University (Haikou, China). The trial areas had no history of the use of the three pesticides. A 30-m^2^ zone (10 m × 3 m) was used as a treatment plot, and another 30-m^2^ zone was defined as a control according to the guidelines of the pesticide analytical method of China^19^. A 5-m^2^ protective barrier was established between the plots. Each treatment and control (sprayed with water) were designed with three replicates. The three pesticides were applied as a spray in the single and combined treatments. The application concentration of each pesticide product was two times the dose recommended by the company (60 ml/ha for avermectin treatment, 40 ml/ha for imidacloprid treatment, and 200 g a.i./ha for carbendazim treatment). Each dose was sprayed three times at an interval of 10 d for the single and combined treatments. Representative soil samples were collected at 2 h and 1, 2, 3, 7, 10, 14, 21, and 28 days after the first application. Additional soil samples were collected 14 days after the second and third application. All soil samples were stored at − 20 °C until use.

### Sample preparation and extraction

Ten grams of soil were put into a 100-mL centrifuge tube, to which 30 mL acetonitrile was added and vortexed for 2 h. Then, 4 g of anhydrous MgSO_4_ and 2 g of NaCl were put into the tube, followed by shaking for 2 min. The tube was centrifuged at 4000 r/min for 5 min. After that, an aliquot of the upper organic layer was collected and dried by a rotary evaporator. The extract residues were redissolved in 2 mL of methanol and filtered with a 0.22-μm nylon syringe filter for high-performance liquid chromatography (HPLC) analysis.

### HPLC analysis

Chromatographic separation was performed on a Waters e2695 HPLC (Waters Associates, USA) with a TechMate C18-ST (5 μM, 4.6 × 250 mm) column (TechMate Technology Co., Ltd.). To detect avermectin, imidacloprid and carbendazim, methanol, water and acetonitrile were used as mobile phase A, mobile phase B and mobile phase C, respectively. A ternary solvent system in gradient mode was operated with a flow rate of 1 mL/min. The linear mobile phase gradient started at 35% A, 45% B and 20% C (0–8 min) and was then maintained at 95% A and 5% B (8–18 min). The column was kept at 30 °C with the photodiode array detection at 245 nm.

### Validation and calculation method

The method linearity was evaluated with a series of calibration standards. Ten microliters of the working standard solutions were injected into the HPLC system, and elution was carried out as described above. The calibration curves were prepared by plotting the peak area versus the concentration of the standard compound. The reproducibility of the method was determined by the intra- and interday precisions. The intra- and interday relative standard deviations (RSDs) were calculated with the pesticides spiked at three different concentrations (0.2, 0.5 and 1 mg/kg) in soil. The limit of quantification (LOQ) was selected at the concentrations that produced a signal-to-noise ratio of 10. The dissipation of the three pesticides follows the first-order exponential decay equation. The degradation half-life values were calculated with the equations C_t_ = C_0_^e−kt^ and t_1/2_ = ln2/k, where C_0_ is the initial concentration, t_1/2_ is the half-life, and C_t_ is the pesticide concentration at time t.

### Acute toxicity test

The artificial soil used for the acute toxicity test consisted of 10% ground sphagnum peat (< 0.5 mm), 20% kaolinite clay (> 45% kaolinite) and 70% fine sand^18^. The desired concentration of the pesticide was dissolved in 10 mL acetone, mixed with 10 g quartz sand for 1 h, and then mixed with the premoistened artificial soil. Approximately 0.65 kg of soil (including 0.5 kg dry artificial soil and 150 mL distilled water) was placed in a 1000-mL beaker, and ten adult earthworms were added to each beaker. The controls were prepared using only 10 mL of water or acetone, both of which did not contain any pesticides. Each treatment was performed in three replicates. To obtain the LC_50_ value of each single pesticide, six dilutions with a geometric ratio were designed for each pesticide within their binary and ternary combinations. To detect interactions within their mixtures, we employed the tested ratios of 1:1 (50% of the LC_50_ value for each pesticide) for binary mixtures and 1:1:1 (33% of the LC_50_ value for each pesticide) for ternary mixtures. Table S1 shows all test concentrations of each pesticide under the individual and combined applications. Under 800 lx of constant light, the beakers were covered with gauze lids and stored at 20 °C with 85% relative humidity. Mortality rates were measured at 14 days after the treatments. The mixture toxicities were predicted by the CI model^20^. According to previous studies, the dose–effect curve parameters and CI values of the three pesticides and their mixtures were computed using CompuSyn software^21,22^.

### Enzyme activity assays

The enzyme activity test was performed according to the method described by Chen et al.^23^. Briefly, the exposure concentration of each pesticide was 10% of the LC_50_ value of the acute toxicity of the artificial soil test. The joint toxicity concentration of three pesticides was conducted at an equal-toxic proportion (1:1:1), which was set as 3.3% of the LC_50_ value of each pesticide in the mixture. The exposure method of the earthworm *E. fetida* was the same as the artificial soil test. After 14 days of exposure, three earthworms were collected, rinsed with distilled water and then weighed. The earthworms were homogenized manually in a vitreous tissue homogenizer with 5 ml phosphate buffer (pH 7.4) and were centrifuged at 3000 r/min for 10 min at 4 °C. The supernatants were collected to determine protein contents and enzyme activities. The protein concentration was measured using a Bradford protein assay kit (Sangon Biotech Co., Shanghai, China). Superoxide dismutase (SOD) activity was determined by inhibiting the photochemical reduction of nitroblue tetrazolium (NBT) as described by Beauchamp and Fridovich^24^. One unit was considered the amount of enzyme that inhibited NBT reduction by 50%, and the results were expressed as U per mg protein. The catalase (CAT) activity was determined by the rate of decomposition of H_2_O_2_, which was evaluated by the decrease in absorbance at 240 nm and expressed as U per mg protein^25^.

### Avoidance test

The avoidance test was designed according to ISO guidelines^26^. Five tested concentrations were prepared based on the LC_50_ values from the acute tests (Tables S2, S3). All test treatments were conducted with three replicates. A two-choice chamber was used for the test. One half of the chamber was filled with 300 g of treated soil, and the other half was filled with 300 g of control soil. Ten earthworms were then placed in the middle of the chamber. The test was performed in the dark for 48 h at 20 °C. Then, the divider was reinserted, and the number of individuals on each side of the container was recorded. Individuals found in the middle of the sections were counted as 0.5 for each side. The avoidance rate A (%) was calculated according to the equation (A = [(C − T)/10] × 100, where C = earthworms observed in the control soil and T = earthworms observed in the treated soil).

### Statistical analysis

Statistical analysis was conducted using the statistical software package SPSS version 13.0 (SPSS, Inc., Chicago, IL, USA). The differences between the pesticide-treated and control groups were determined with Student’s t test and Fisher’s least significant difference (LSD) test. P values below 0.05 were considered statistically significant.

## Results

### Assay validation

The residual analysis method for avermectin, imidacloprid and carbendazim in soil was established and validated. The baseline separation of the three pesticides was determined with an HPLC system containing a C18 column (Fig. S1). Good linearity was achieved within the concentration range of 0.02–20 mg/L with linear equations y = 22804x + 50.46 (R^2^ = 0.9997), y = 51339x + 706.75 (R^2^ = 0.9993), and y = 26858x + 108.92 (R^2^ = 0.9995) for avermectin, imidacloprid, and carbendazim, respectively. The LOQ values for these pesticides in soil were all below 0.01 mg/kg. The average recoveries of the three pesticides from soils were between 80–104% with relative standard deviations of 2.0–10.1% (Table S4), indicating repeatability of the method.

### Degradation of the binary and ternary mixtures of the three pesticides in soil

**Figure 1:**
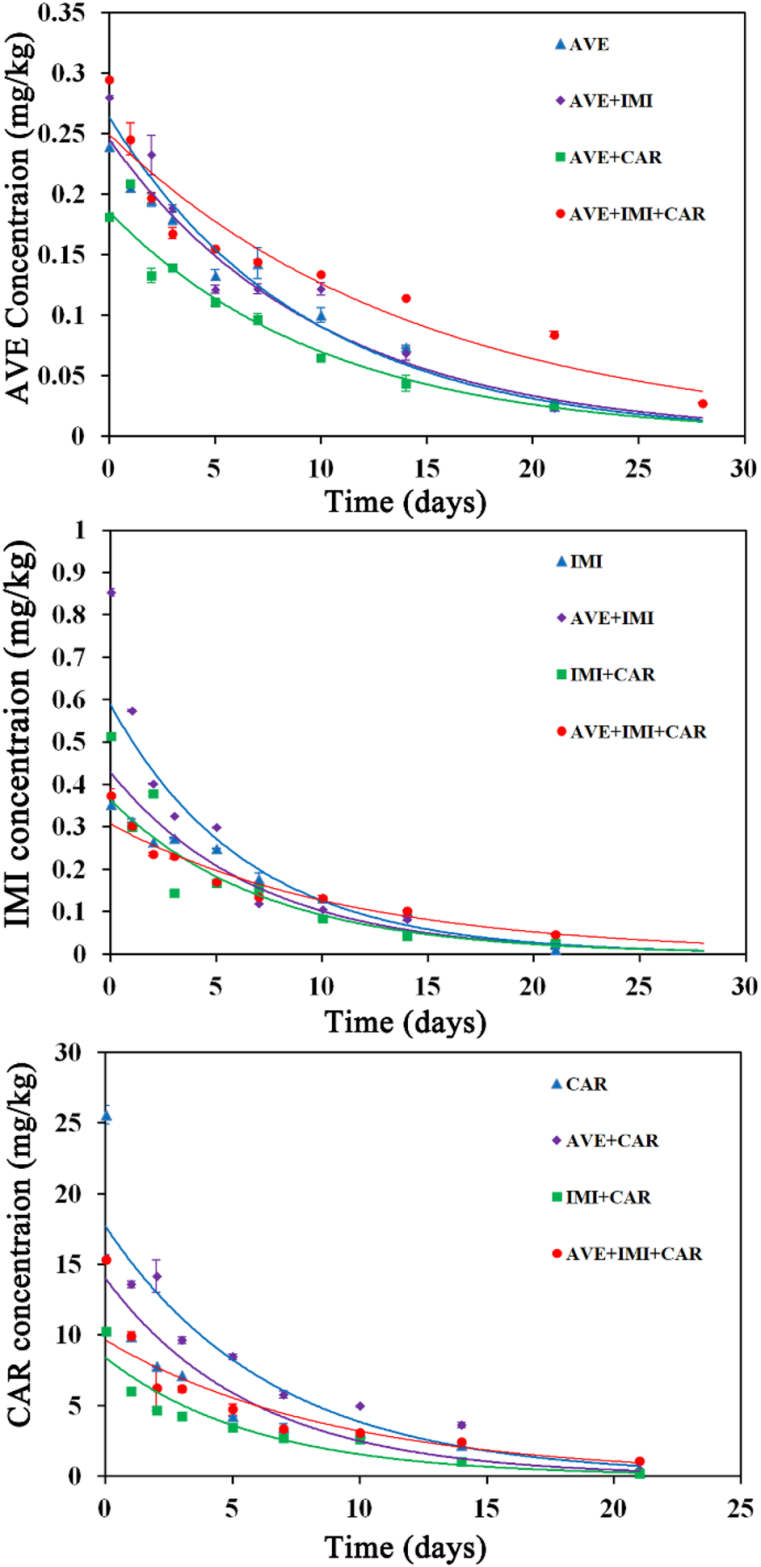
Dissipation of avermectin (AVE), imidacloprid (IMI) and carbendazim (CAR) individually and combined in soil. Solid lines show the first-order kinetics results. Values are expressed as the means ± standard errors (SEs) of three replicates.

**Figure 2:**
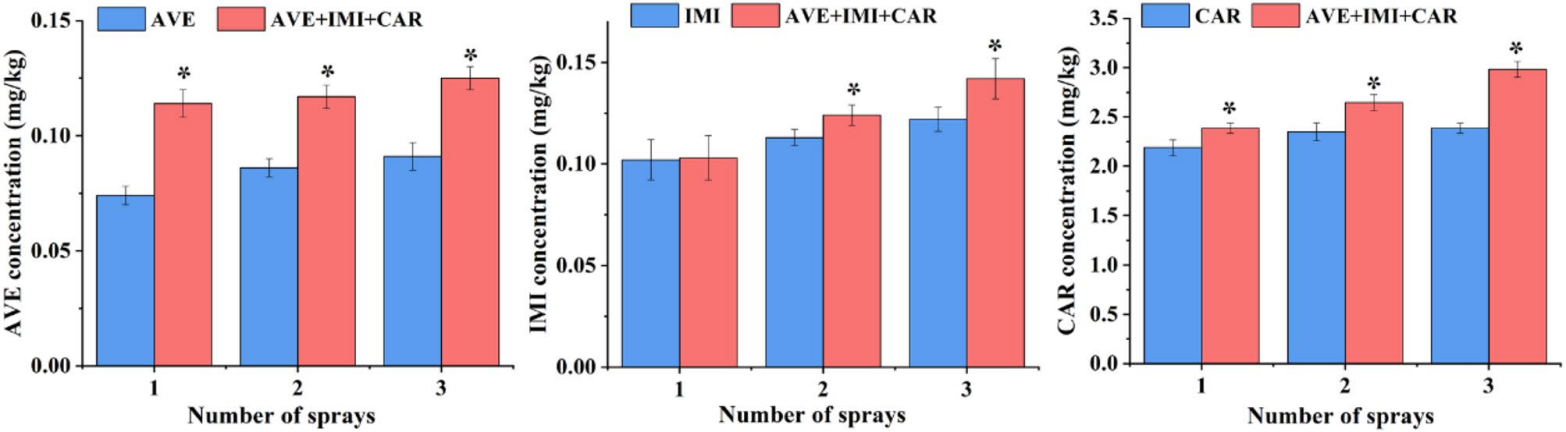
Residues of avermectin, imidacloprid and carbendazim used individually and in combination in soil. Soil samples were collected 14 days after two or three applications of each treatment. Values are expressed as the means and SEs of three replicates. Statistical analyses were performed using Student’s t test: *P < 0.05.

As shown in Fig. 1 and Table 1, the dissipation of avermectin, imidacloprid, carbendazim and their mixtures in soils followed first-order kinetics with high correlation coefficients (R^2^ ≥ 0.9132). The degradation half-life values of the pesticides ranged from 4.10 to 10.19 days under the individual and combined applications. Compared with the DT50 the single pesticide, the DT50 values of avermectin, the DT50 values of these pesticides in the binary mixtures were similar. However, their DT50 values in the ternary mixtures were approximately 1.5 times higher than those in the single-use group. We further applied solutions of avermectin, imidacloprid, carbendazim and their ternary mixture either two or three times in soil, and the pesticide residue amounts at 14 days after application are shown in Fig. 2. The amount of residues of avermectin, imidacloprid and carbendazim in soil with a single application were 0.086–0.091 mg/kg, 0.113–0.122 mg/kg and 2.350–2.386 mg/kg, respectively. The amount of residues of avermectin, imidacloprid and carbendazim in soil with mixed application were 0.117–0.125 mg/kg, 0.124–0.142 mg/kg and 2.646–2.982 mg/kg, respectively. The amount of residues of these pesticides after mixed application were significantly higher than those after application alone.

**Table 1:** Degradation parameters of the three pesticides used individually or in combination in soil.

Chemicals	Treatments^a^	Resolution equation	R^2^	DT50 (days)	Fold change^b^
Avermectin (AVE)	AVE	C_t_ = 0.2455e^−0.100t^	0.9668	6.93	–
	AVE + IMI	C_t_ = 0.2639e^−0.107t^	0.9511	6.48	0.94
	AVE + CAR	C_t_ = 0.1856e^−0.098t^	0.9767	7.07	1.02
	AVE + IMI + CAR	C_t_ = 0.2495e^−0.068t^	0.9143	10.19	1.47
Imidacloprid (IMI)	IMI	C_t_ = 0.4295e^−0.144t^	0.9001	4.81	–
	AVE + IMI	C_t_ = 0.5887e^−0.154t^	0.9432	4.50	0.94
	IMI + CAR	C_t_ = 0.3641e^−0.138t^	0.9202	5.02	1.04
	AVE + IMI + CAR	C_t_ = 0.3072e^−0.089t^	0.9562	7.79	1.62
Carbendazim (CAR)	CAR	C_t_ = 13.997e^−0.173t^	0.9152	4.01	–
	AVE + CAR	C_t_ = 17.683e^−0.153t^	0.9336	4.53	1.13
	IMI + CAR	C_t_ = 8.3956e^−0.169t^	0.9452	4.10	1.02
	AVE + IMI + CAR	C_t_ = 9.6272e^−0.110t^	0.9132	6.30	1.57

^a^The three pesticides were applied as a foliar spray in the single and combined treatments.

^b^Fold change = Half-lives combined used in soil/ half-lives individual used in soil.

### Joint acute toxicity of the three pesticides to the earthworm *E. fetida*

**Figure 3:**
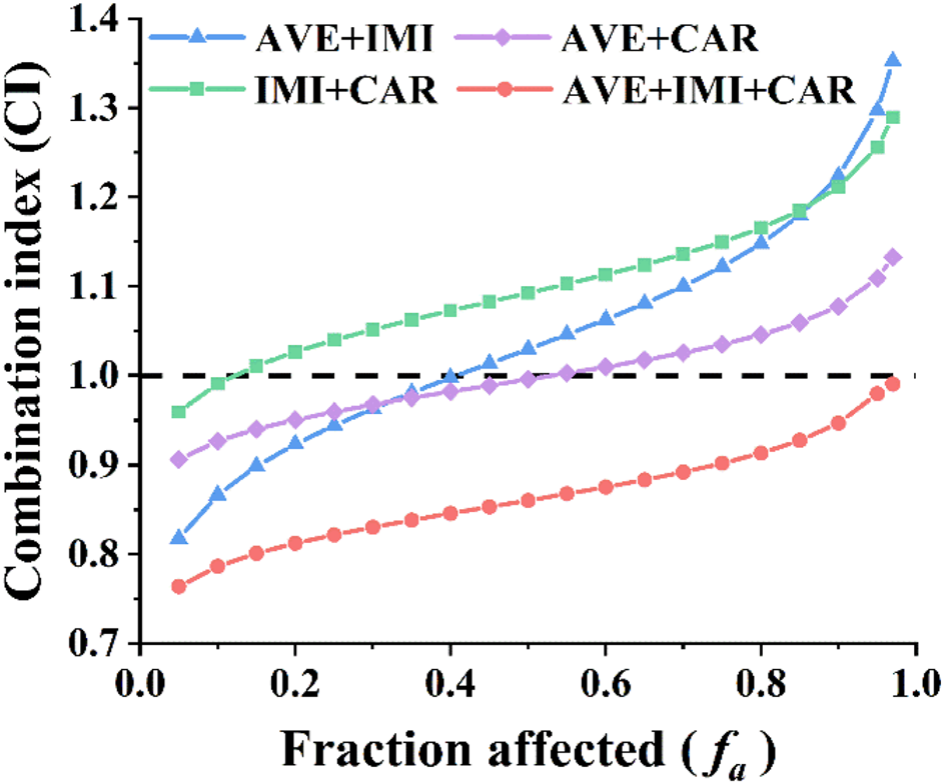
Combination index plot for binary and ternary mixtures of the three pesticides in the artificial soil acute toxicity test. CI values are plotted as a (*f*_a_) of the earthworms by computer simulation. CI < 1, = 1 and > 1 indicates synergism, an additive effect and antagonism, respectively.

The CI-isobologram method was used to determine the nature of the toxicological interactions among avermectin, imidacloprid and carbendazim toward the earthworm *E. fetida* by the acute toxicity test. Table 2 shows the dose–effect curve parameters (Dm, m, and r) of the individual pesticides and their mixtures, as well as the mean CI values of the mixtures. Dm is the median lethal concentration and equals the LC_50_ value^26^. The Dm values of avermectin, imidacloprid and carbendazim were 26.83, 3.18 and 8.46 mg/kg, respectively. All dose–effect curves were sigmoidal (m > 1) and conformed to the median effect principle (r > 0.93). CI values plotted as a function of the mortality rate (*f*_a_) shows the types of interaction (synergism, antagonism and additive effect) as a function of the level of the effect of pesticide mixtures toward *E. fetida* (Fig. 3). The binary mixtures of the pesticides showed changing interactions throughout the entire effect level range. In contrast, stable synergistic responses were noted for their ternary mixtures with CI values less than 1.

**Table 2:** Dose–effect parameters and CI values of the three pesticides and their binary and ternary mixtures on acute toxicity toward *Eisenia fetida*.

Chemicals and mixtures (ratio)	Dose–effect parameters^a^			CI values^b^					
	Dm (mg/kg)	m	R	LC_10_		LC_50_		LC_90_	
Avermectin (AVE)	26.83	10.43 ± 0.8	0.9396						
Imidacloprid (IMI)	3.18	9.04 ± 0.33	0.9595						
Carbendazim (CAR)	8.46	5.64 ± 0.62	0.9826						
AVE + IMI (1:1)	15.45	5.50 ± 0.35	0.9960	0.87	Syn	1.03	Add	1.22	Ant
AVE + CAR (1:1)	17.55	5.85 ± 0.66	0.9816	0.93	Syn	1.00	Add	1.08	Ant
IMI + CAR (1:1)	6.36	5.27 ± 0.36	0.9906	0.99	Add	1.09	Ant	1.21	Ant
AVE + IMI + CAR (1:1:1)	11.02	5.87 ± 0.36	0.9944	0.79	Syn	0.86	Syn	0.95	Syn

^a^The parameters Dm, m, and r represent the potency (LC_50_), the linear correlation coefficient of the median-effect plot, and the accordance of data to the mass-action law, respectively.

^b^CI < 1, CI = 1, and CI > 1 mean synergism (Syn), additive effect (Add), and antagonism (Ant), respectively. LC_10_, LC_50_ and LC_90_ represent the doses causing 10, 50, and 90% mortality rate of *Eisenia fetida*, respectively.

### Joint toxicity of the three pesticides on the SOD and CAT activities of *E. fetida*

**Figure 4:**
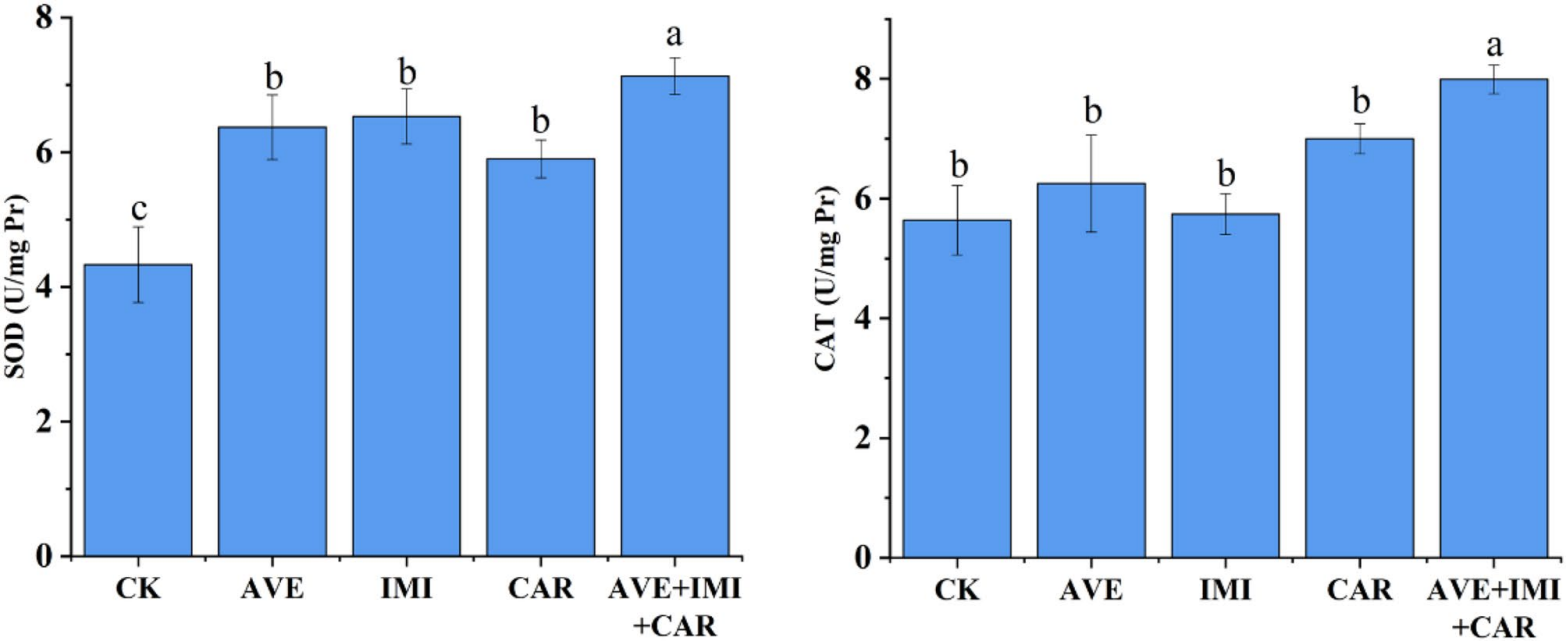
Effects of avermectin, imidacloprid, carbendazim and their ternary mixture on SOD activity and CAT activity in *E. fetida*. Values are the means and SEs of three replicates. Means with different letters are significantly different according to the LSD test (*P* < 0.05).

To further confirm the joint acute toxicity of avermectin, imidacloprid and carbendazim to *E. fetida*, the effects of the three pesticides and their mixtures on two antioxidative enzyme activities of *E. fetida* were investigated, and the results are shown in Fig. 4. SOD activity was activated with the single pesticide, while CAT activity showed no change. In contrast, a considerable increase in SOD and CAT activities was found under the ternary treatment.

### Joint toxicity of the three pesticides on the avoidance behavior of *E. fetida*

**Figure 5:**
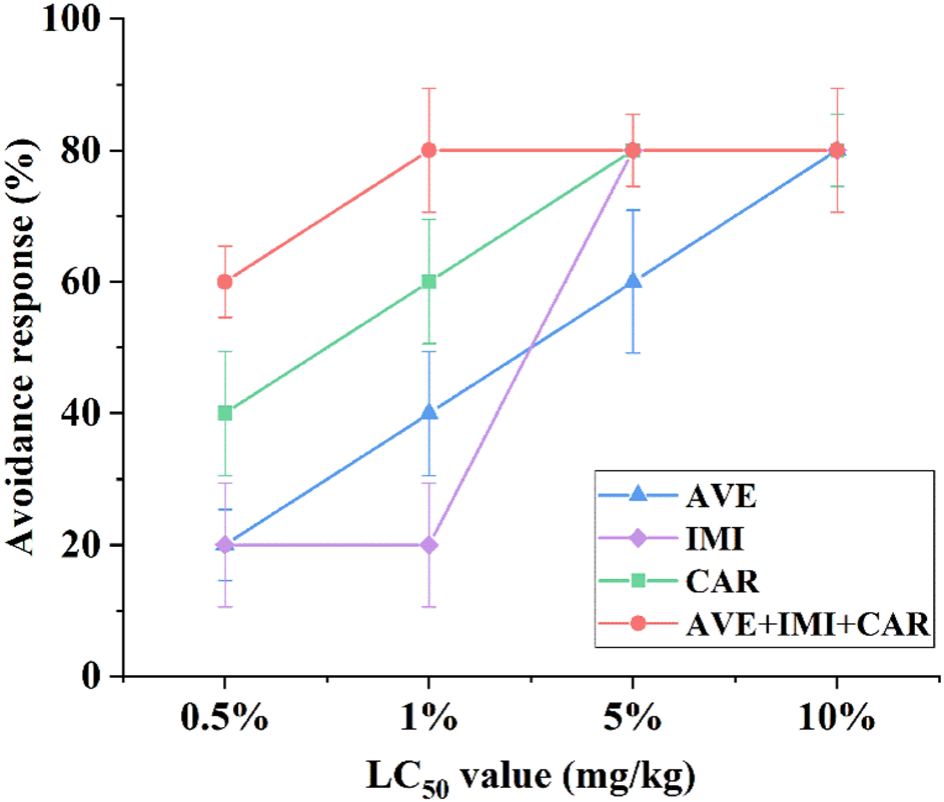
Avoidance responses of *E. fetida* to avermectin, imidacloprid, carbendazim and their ternary mixture. Values are expressed as the means and SEs of three replicates.

To explore a method for assessing joint toxicity within a shorter test period, we evaluated the avoidance behavior of *E. fetida* in response to the three pesticides and their ternary mixture. Our results showed a significant difference between individual and combined applications at LC_50_ values of 0.5% and 1%. There was a positive correlation between the toxicity of these pesticides and the avoidance behavior of *E. fetida*. The number of individuals observed in the control soil was greater than 80% in the treatment, which means that this soil has a limited habitat function and is unsuitable for this species^27^. The concentrations of avermectin, imidacloprid, carbendazim, and their ternary mixture were 10%, 5%, 5%, and 1% of the LC_50_ values, respectively, with approximately 80% of the individuals avoiding the treatment (Fig. 5). The avoidance effects increased considerably under combined treatments of the three pesticides compared to the individual treatments.

## Discussion

Mixtures of pesticides in agricultural fields are common. Insecticides and fungicides are widely applied together as a combined pesticide to control insect pests and plant diseases simultaneously^28^. The current risk assessment is based on a single chemical, which is not effective in evaluating its environmental behavior and ecotoxicology in the natural system because of the compound interaction in a mixture system^29^. In this study, we assessed the effects of three common pesticides through mixed application on soil degradation and toxicity towards earthworm.

Degradation is an important environmental behavior of pesticides in soil, which is directly related to the residence time and the degree of impact on the soil environment^30^. In this study, we investigated the dissipation dynamics and residues of avermectin, imidacloprid and carbendazim and compared the differences between their individual and combined applications. All dissipation of the three pesticides and their binary and ternary mixtures in soils followed first-order kinetics. The degradation half-life values of the single and mixed pesticides were not higher than 10.19 days. According to the Chinese National Standard GB/T 31270.1-2014, all these pesticides are easily degraded pesticides (half-life values < 30 d) under both individual and combined applications. However, the DT50 values of the three pesticides in the ternary mixtures increased considerably compared with those in the individual application, which led to higher residues in soil after several applications. This is in accordance to the scientific literature, which states that mixed application prolongs the degradation time of certain pesticides in soil. For example, the fungicide chlorothalonil significantly inhibits the degradation of the herbicide metolachlor in soil, and the inhibition rate is up to two times^31^. The fungicide mancozeb and its mixture with the insecticide thiamethoxam significantly suppress the degradation of the herbicide pendimethalin in soil^2^. A possible reason may be that microbial degradation of a compound in a mixture can be strongly impacted by other compounds in the mixture^32,33^. Thus, the combined application of pesticides might suppress the degradative activity of soil microorganisms. Our hypothesis is that the ternary mixture of avermectin, imidacloprid and carbendazim significantly hampers the survival of essential microorganisms responsible for degrading these pesticides, leading to a substantial decline in pesticide degradation efficiency. However, the underlying mechanism still needs further exploration.

Earthworms are often regarded as a key indicator species for ecotoxicological assessments of soil pollution because of their high biomass and ecological importance in soil^34^. To obtain information about the ecotoxicological effects of avermectin, imidacloprid and carbendazim in soil, the earthworm *E. fetida* was exposed to artificial soil containing each pesticide or their mixture, and the acute toxicity was measured. The LC_50_ values of the three pesticides are consistent with previous reports, which showed that avermectin was classified as low toxic, while imidacloprid and carbendazim were classified as moderately toxic^10,12^. However, some reports indicate that imidacloprid is highly toxic to earthworms and is prohibited for both seed coating and soil treatment within the European Union^35,36^. The LC_50_ values of their binary and ternary mixtures fall between the maximum and minimum LC_50_ values of the three pesticides, indicating that the combined use balances their respective toxicities. The CI equation method has been widely used to calculate the predictions of mixture toxicity^15,20,37^. In this study, the joint toxicity of the three pesticides was determined with the CI model and visualized by isobolograms. The ternary mixture of the pesticides showed stable synergistic behavior throughout the entire effect level range. Thus, these multicomponent mixtures might pose a larger threat to soil organisms. Two antioxidative enzymes, SOD and CAT, have been used as important biochemical biomarkers of exposure to pollutants that trigger oxidative stress^38^. We evaluated the toxicity of the ternary mixture towards *E. fetida* by measuring the enzyme activities. The ternary mixture elicited a significant increase in SOD and CAT activities compared to individual treatments at equitoxic concentrations. The enzyme test indicated that treatment with the ternary mixtures had more adverse effects on the survival of *E. fetida* than the individual treatments. These results also indicated that the co-occurrence of these pesticides caused more negative effects on the soil organisms than expected. In recent years, a growing body of research has shown that the toxicity effects of pesticide mixtures on nontarget organisms are synergistic^39–41^. The predicted synergism in most pesticide mixtures at low-effect levels indicates a potential ecotoxicological risk associated with the co-occurrence of these chemicals even at low concentrations^15^. The synergistic effects of these pesticides should be of great concern to regulatory authorities and the public. It is necessary to reconsider the current procedures for determining the quality of soils and effects of chemicals on behavior, which likely underestimate the negative effects of chemical mixtures.

The earthworm mortality test is frequently used in current standardized laboratory test systems. The test provides information on the toxic effects of contaminants on exposed organisms but does not offer firsthand insight into the reactions and behaviors of these organisms when exposed to contaminants in the soil^42^. Earthworms are also a suitable early warning system due to their rapid response to both natural and anthropogenic stressors^43^. Various chemicals have been studied for toxicity by earthworms using avoidance test^44–46^. In addition, the mortality test requires a longer period and is much more labor-intensive than the avoidance test. Therefore, the avoidance test is a promising candidate for a short-time screening test to determine the joint toxicity of chemicals toward earthworms. In this study, there was a positive correlation between the toxicity of pesticides and the avoidance behavior of *E. fetida* under both individual and combined applications. Some studies have shown that the avoidance behaviors toward pesticides at concentrations in a range are similar to the acute test results^17,47^. Therefore, the avoidance test is useful as a screening tool for the preliminary assessment of pesticides and their mixtures in soils.

## Conclusion

In this study, we determined the effects of mixture interactions of avermectin, imidacloprid and carbendazim on their degradation in soil and toxicity toward *E. fetida*. Their ternary combinations significantly increased the DT50 of each pesticide. The ternary mixtures of these pesticides showed stable synergistic toxicity toward the earthworms. Our findings contribute to understanding the complexity of effects from pesticide mixtures on non-target organisms and provide useful information about the interactions of pesticides in soil.

### Supplementary Information

Supplementary Information.

## Data Availability

It should be justified that “All data generated or analysed during this study are included in this published article [and its supplementary information files]”.
