# Supplementary material for: Effects of mixed application of avermectin, imidacloprid and carbendazim on soil degradation and toxicity toward earthworms
Source: Sci Rep. 2023 Aug 29;13:14115. doi: 10.1038/s41598-023-41206-1 (PMC10465560; doi:10.1038/s41598-023-41206-1)
Supplement: Supplementary file 1 — Supplementary Information. [file 41598_2023_41206_MOESM1_ESM.doc]

**Table S1 The concentrations of each pesticide under the individual and combined applications in acute toxicity tests**

| No. | Concentration (mg/kg) | | | | | | | | | | | | | | | | | | | | | | |
| --- | --- | --- | --- | --- | --- | --- | --- | --- | --- | --- | --- | --- | --- | --- | --- | --- | --- | --- | --- | --- | --- | --- | --- |
|  | Avermectin  (AVE) |  | Imidacloprid  (IMI) |  | Carbendazim  (CAR) |  | AVE + IMI | | |  | AVE + CAR | | |  | IMI + CAR | | |  | AVE + IMI + CAR | | | | |
|  |  |  |  |  |  |  | AVE |  | IMI |  | AVE |  | CAR |  | IMI |  | CAR |  | AVE |  | IMI |  | CAR |
| 1 | 23.62 |  | 2.36 |  | 7.08 |  | 13.40 |  | 1.59 |  | 12.06 |  | 3.82 |  | 1.43 |  | 3.82 |  | 8.04 |  | 0.95 |  | 2.55 |
| 2 | 26.24 |  | 2.62 |  | 7.87 |  | 14.89 |  | 1.77 |  | 13.40 |  | 4.24 |  | 1.59 |  | 4.24 |  | 8.93 |  | 1.06 |  | 2.83 |
| 3 | 29.16 |  | 2.92 |  | 8.75 |  | 16.54 |  | 1.96 |  | 14.89 |  | 4.71 |  | 1.77 |  | 4.71 |  | 9.92 |  | 1.18 |  | 3.14 |
| 4 | 32.40 |  | 3.24 |  | 9.72 |  | 18.38 |  | 2.18 |  | 16.54 |  | 5.23 |  | 1.96 |  | 5.23 |  | 11.02 |  | 1.31 |  | 3.49 |
| 5 | 36.00 |  | 3.60 |  | 10.80 |  | 20.42 |  | 2.42 |  | 18.38 |  | 5.82 |  | 2.18 |  | 5.82 |  | 12.25 |  | 1.45 |  | 3.88 |
| 6 | 40.00 |  | 4.00 |  | 12.00 |  | 22.69 |  | 2.69 |  | 20.42 |  | 6.46 |  | 2.42 |  | 6.46 |  | 13.61 |  | 1.62 |  | 4.31 |

**Table S2 Acute toxicities of the three pesticides, alone or in mixtures, towards *E.fetida***

| Chemicals | Combination (ratio) | Resolution equation | R^2^ | LC_50_ (mg/kg)^a^ |
| --- | --- | --- | --- | --- |
| Avermectin  (AVE) | AVE | Y=14.0040x-15.0010 | 0.8914 | 26.81 |
|  | AVE+IMI (1:1) | Y=7.5331x-3.5942 | 0.9965 | 13.83 |
|  | AVE+CAR(1:1) | Y=7.9116x-3.8937 | 0.9806 | 13.31 |
|  | AVE+IMI+CAR (1:1:1) | Y=7.9939x-2.0715 | 0.9948 | 7.67 |
| Imidacloprid  (IMI) | IMI | Y=12.2381x-1.1471 | 0.9157 | 3.18 |
|  | AVE+IMI (1:1) | Y=7.5331x+3.3792 | 0.9965 | 1.64 |
|  | IMI+CAR (1:1) | Y=7.2324x+3.2724 | 0.9916 | 1.73 |
|  | AVE+IMI+CAR (1:1:1) | Y=7.9948x+5.3272 | 0.9948 | 0.91 |
| Carbendazim  (CAR) | CAR | Y=7.7814x-2.2232 | 0.9722 | 8.48 |
|  | AVE+CAR (1:1) | Y=7.9116x+0.0600 | 0.9806 | 4.21 |
|  | IMI+CAR (1:1) | Y=7.2325x+0.1915 | 0.9916 | 4.62 |
|  | AVE+IMI+CAR (1:1:1) | Y=7.9947x+1.9176 | 0.9948 | 2.43 |

^a^ LC_50_, the effective concentration that results in a 50% reduction in population growth compared to the control.

**Table S3** The concentrations of each pesticide under the individual and combined applications in avoidance test

| Combination (ratio) | Chemicals | 14d-LC_50_  (mg/kg) | Concentration (mg/kg) | | | | | | | |
| --- | --- | --- | --- | --- | --- | --- | --- | --- | --- | --- |
|  |  |  |  | 0.5% LC_50_ |  | 1% LC_50_ |  | 5% LC_50_ |  | 10% LC_50_ |
|  |  |  |  |  |  |  |  |  |  |  |
|  | Avermectin  (AVE) | 26.81 |  | 0.13 |  | 0.27 |  | 1.34 |  | 2.68 |
|  | Imidacloprid  (IMI) | 3.18 |  | 0.02 |  | 0.03 |  | 0.16 |  | 0.32 |
|  | Carbendazim  (CAR) | 8.48 |  | 0.04 |  | 0.08 |  | 0.42 |  | 0.85 |
| AVE+IMI (1:1) | AVE | 13.83 |  | 0.07 |  | 0.14 |  | 0.69 |  | 1.38 |
|  | IMI | 1.64 |  | 0.01 |  | 0.02 |  | 0.08 |  | 0.16 |
| AVE+CAR(1:1) | AVE | 13.31 |  | 0.07 |  | 0.13 |  | 0.67 |  | 1.33 |
|  | CAR | 4.21 |  | 0.02 |  | 0.04 |  | 0.21 |  | 0.42 |
| IMI+CAR (1:1) | IMI | 1.73 |  | 0.01 |  | 0.02 |  | 0.09 |  | 0.17 |
|  | CAR | 4.62 |  | 0.02 |  | 0.05 |  | 0.23 |  | 0.46 |
| AVE+IMI+CAR (1:1:1) | AVE | 7.67 |  | 0.04 |  | 0.08 |  | 0.38 |  | 0.77 |
|  | IMI | 0.91 |  | 0.005 |  | 0.01 |  | 0.05 |  | 0.09 |
|  | CAR | 2.43 |  | 0.01 |  | 0.02 |  | 0.12 |  | 0.24 |

**Table S4** Recovery and RSD of avermectin, imidacloprid and carbendazim in soil at different spiked levels

| Spiked compound | Spiked  level  (mg/kg) |  |  | | Intraday（n=5） | | | | | |  | Unterday（n=15） |
| --- | --- | --- | --- | --- | --- | --- | --- | --- | --- | --- | --- | --- |
|  |  | Day 1 | |  | | Day 2 | |  | Day 3 | |  |  |
|  |  | recovery  (%) | RSD  (%) |  | | recovery (%) | RSD (%) |  | recovery（%） | RSD（%） |  | RSD（%） |
| Avermectin | 0.2 | 89±3.6 | 10.1 |  | | 96±4.8 | 12.5 |  | 95±3.4 | 8.8 |  | 4.0 |
|  | 0.5 | 80±2.0 | 6.2 |  | | 84±1.5 | 4.5 |  | 83±2.0 | 5.9 |  | 3.0 |
|  | 1 | 83±3.2 | 9.8 |  | | 81±1.8 | 5.8 |  | 88±1.3 | 4.1 |  | 3.4 |
| Imidacloprid | 0.2 | 97±2.4 | 6.1 |  | | 95±1.3 | 3.4 |  | 93±1.6 | 4.3 |  | 1.9 |
|  | 0.5 | 97±1.9 | 5.0 |  | | 92±2.2 | 5.8 |  | 94±1.1 | 2.8 |  | 2.6 |
|  | 1 | 104±2.3 | 5.6 |  | | 97±2.0 | 5.0 |  | 97±1.4 | 3.5 |  | 4.1 |
| Carbendazim | 0.2 | 82±1.9 | 5.8 |  | | 86±2.1 | 6.6 |  | 82±1.8 | 5.5 |  | 2.0 |
|  | 0.5 | 98±1.2 | 4.0 |  | | 87±1.3 | 4.2 |  | 101±1.3 | 4.2 |  | 2.4 |
|  | 1 | 85±1.9 | 5.7 |  | | 81±1.6 | 5.0 |  | 83±1.4 | 4.3 |  | 3.3 |

Values are the means and standard deviations (SDs) of five replicates.


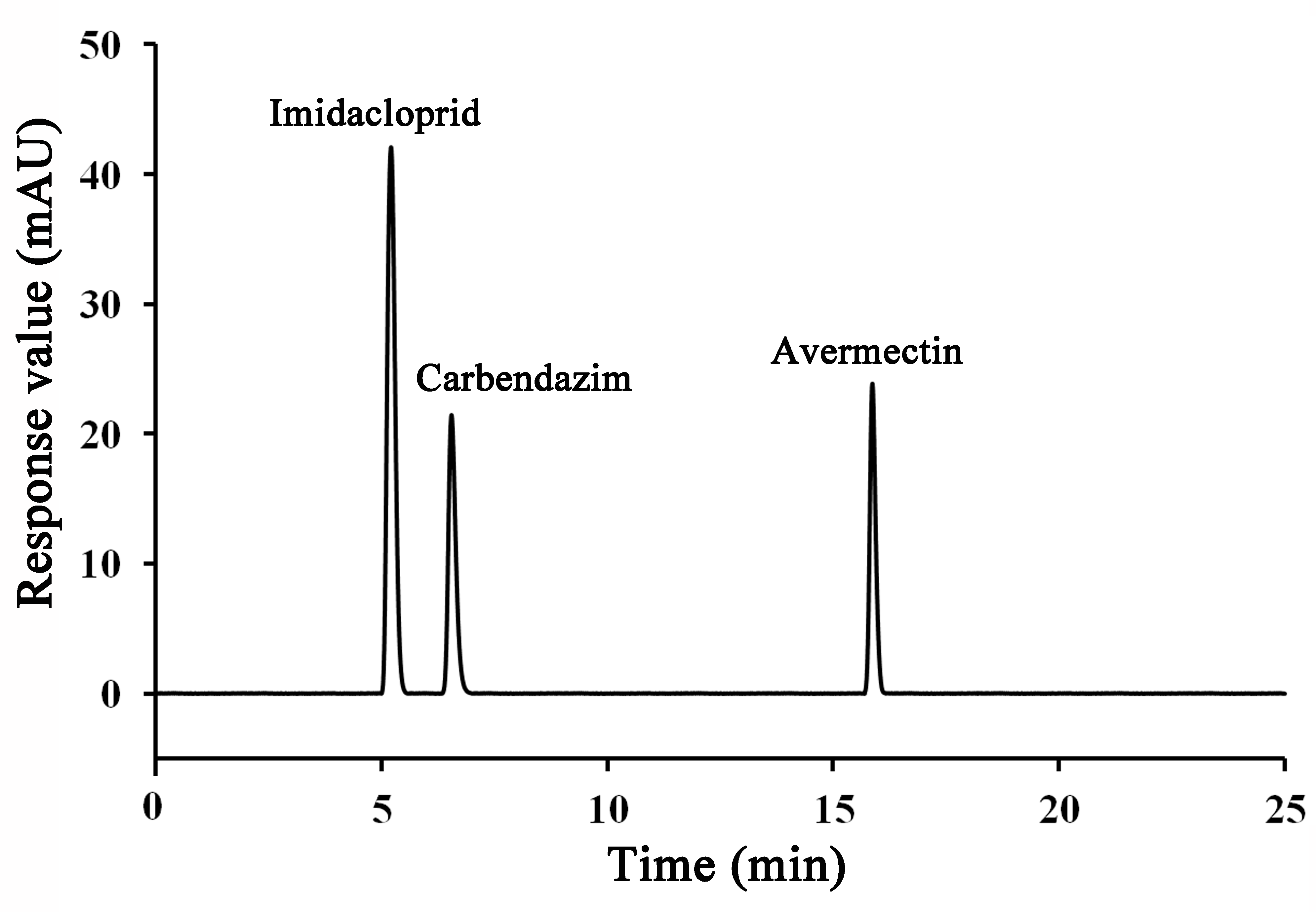


**Fig. S1** LC chromatogram of avermectin, imidacloprid and carbendazim
